# Differential Gene Expression between Limbal Niche Progenitors and Bone Marrow Derived Mesenchymal Stem Cells

**DOI:** 10.7150/ijms.40881

**Published:** 2020-02-10

**Authors:** Wei Wang, Shen Li, Lingjuan Xu, Menglin Jiang, Xinyu Li, Yuan Zhang, Sean Tighe, Yingting Zhu, Guigang Li

**Affiliations:** 1 Department of Ophthalmology, Tongji Hospital, Tongji Medical College, Huazhong University of Science and Technology, Wuhan, Hubei Province 430030, China; 2 Tissue Tech Inc, Miami, Florida, 33126 USA

**Keywords:** Gene expression, gene chip, LNC, BMMSC

## Abstract

**Purpose**: To compare the difference in gene expression between human limbal niche cells (LNC) and bone marrow derived mesenchymal stem cells (BMMSC).

**Methods**: LNC were isolated by collagenase and expanded in modified embryonic stem cell medium (MESCM) on a Matrigel coated plastic plate. Cell diameters were measured with Image J software. Relative gene expression levels between LNC and BMMSC were compared using Affymetrix Human Primer View Gene Expression Array. A subset of differentially expressed genes was verified by RT-qPCR. The protein level of LAMA1 and COL4A1 was confirmed by Western blot and immunostaining.

**Results**: The average diameter of LNC was 10.2±2.4 μm, which was significantly smaller than that of BMMSC (14 ±3.4 μm) (p<0.0001). Expression of 20,432 genes was examined by Gene Expression Array, among which expression of 349 genes in LNC was 10-fold or higher than that of BMMSC and expression of 8 genes in LNC was 100-fold or higher than that of BMMSC, while expression of 3 genes in BMMSC was 100-fold higher than that of LNC. GO analysis and pathway analysis showed that the differentially expressed genes were mainly enriched in the extracellular matrix receptor interaction pathway and Wnt signaling pathway. In addition, RT-qPCR results demonstrated that the expression of CD73, CD90, CD105, PDGFRβ, Vimentin, SCF, KIT (CD117), COL14A1, LAMA2, THBS2, FZD1, BMP2 and CXCL12 genes in LNC were at least 2 folds higher than BMMSC. The protein level of LAMA1 was higher but the protein level of COL4A1 was lower in LNC than that in BMMSC.

**Conclusion**: LNC exhibit differential gene expression from BMMSC in the extracellular matrix (ECM) receptor interaction pathway and Wnt signaling pathway, suggesting that LNC have their unique signaling pathways to support limbal stem cell niches.

## Introduction

Limbal niche cells (LNC) are a group of primitive cells isolated from the microenvironment around the limbal stem cells (LSC) from cornea. LNCs are capable of generating progenitor cells with angiogenesis and mesenchymal stem cells (MSC) properties and support limbal epithelial progenitor cells (LEPC) in vitro under 3D Matrigel culture condition 1-4. In vitro cultured LNCs adhere to Matrigel coated plastic plates and have a spindle shape with increased expression of stromal marker Vimentin but not epithelial marker PCK 1-4. LNC heterogeneously express stem cell markers such as OCT4, SOX2, NANOG, REX1, NESTIN, N-CARHERIN, SSEA4, and CD34, as well as MSC markers such as CD73, CD90 and CD105 1-4. In addition, LNC can be differentiated into osteoblasts, adipocytes and chondroblasts *in vitro* 1-4. Injected subconjunctivally or systematically, LNC have been shown to promote wound healing after corneal alkali burn and prevent limbal stem cell deficiency (LSCD) in rats or rabbits, as demonstrated by increased corneal transparency and decreased fluorescein staining and neovascularization 5. Although both LNC and BMMSC are able to facilitate the healing of the cornea after alkaline burn, the efficacy of LNC is higher than that in BMMSC 6.

BMMSC are well known for their pluripotency and potential applications in many serious diseases, including articular cartilage defects, Crohn's disease, acute myocardial infarction, post-myocardial infarction, chronic myocardial ischemia, steroid-resistant acute graft-versus-host disease, organ transplantation, liver fibrosis, type I diabetes mellitus and multiple sclerosis 7. BMMSC are well defined as plastic-adherent cells positive for CD73, CD90, CD105, while negative for CD11b, CD14, CD19, CD34, CD45, CD79a or HLA-DR surface molecules and can be differentiated into osteoblasts, adipocytes and chondroblasts in vitro 8. BMMSC have also been shown to differentiate into corneal epithelial like cells 9, 10 and keratocytes 11, 12. In fact, BMMSC transplantation has been shown to promote cornea wound healing after alkali burn 9, 10, 13-16 and facilitate the regeneration of the corneal stroma after penetrating injury 12. These studies suggest that BMMSC could be used to prevent or treat LSCD as a novel cell therapy tool.

Previously, we have shown LNC can maintain the stemness of LEPCs better than BMMSC when co-cultured in 3D Matrigel, both of which could form spheres after ten days of culture, although LEPC co-cultured with LNC expressed higher level of p63α but lower level of CK12 1, 2. Animal experiments verified these observations wherein both subconjunctivally injected LNCs and BMMSCs prevented LSCD caused by alkali burn, yet LNC treated corneas had less corneal opacity and faster epithelial healing 17. Although these results suggest LNC are therapeutically more advantageous than BMMSC for LSCD, the mechanism remains unclear.

In this study, we examined the differential gene profiles between LNCs and BMMSCs when they serve as LSC niche cells using a whole genome human gene expression microarray.

## Materials and Methods

### Cell Isolation and Culture

This study was approved by the ethical committee of Tongji Hospital. Human LNC were isolated and cultured as previously reported 1, 2. Corneoscleral rims were obtained from the Red Cross Eye Bank of Wuhan City, Tongji Hospital (Hubei, China) and managed in accordance with the Declaration of Helsinki. The limbal explants were cut into 12 average sections and digested with collagenase A (Coll) at 37 °C for 10 hours to generate clusters containing the limbal epithelial sheet and subjacent LNC. The clusters were digested further with 0.25% trypsin and 1 mM EDTA (T/E) at 37 °C for 15 minutes to yield single cells before being seeded at the density of 1×10^4^ per cm^2^ in 6-well plates coated with Matrigel in modified embryonic stem cell medium (MESCM). BMMSC (HUXMA-01001, Cyagen, Guangzhou, China) were cultured in a similar manner as control. Upon 80% confluence, cells were passaged serially with T/E at the density of 5 ×10^3^ per cm^2^, and the 4^th^ passage cells were used for the following experiments. All materials used for cell isolation and culturing are listed in Supplementary materials Table S1.

### Immunofluorescence Staining

Single cells were prepared for cytospin using Cytofuge at 1000 rpm for 8 minutes (StatSpin, Inc.), fixed with 4% formaldehyde for 15 minutes, permeabilized with 0.2% Triton X-100 in PBS for 15 minutes, and blocked with 2% BSA in PBS for 1 hour before being incubated with primary antibodies overnight at 4°C. After washing with PBS, cytospin preparations were incubated with corresponding secondary antibodies for 1 hour using appropriate isotype-matched nonspecific IgG antibodies as controls. The nuclei were counter-stained with Hoechst 33342 before being analyzed with Zeiss LSM 700 confocal microscope (LSM700; Carl Zeiss). Detailed information about primary and secondary antibodies and agents used for immunostaining are listed in Supplementary Table S1.

### Western Blot

Proteins were extracted by RIPA buffer supplemented with proteinase inhibitors and phosphatase. The protein concentration was determined by a BCA protein assay (Pierce, Rockford, IL). Equal amounts of proteins in total cell extracts were separated by 8% SDS-PAGE and transferred to nitrocellulose membranes that were then blocked with 5% (wt/vol) fat-free milk in TBST (50 mM Tris-HCl, pH 7.5, 150 mM NaCl, 0.05% (vol/vol) Tween-20), followed by sequential incubation with specific primary antibodies and their respective secondary antibodies using β-actin as the loading control. Immunoreactive bands were visualized by a chemiluminescence reagent (Western Lighting; Pierce). Antibodies used are listed in Supplementary Table S1.

### Cell Diameter Measurement

A total of 200μl cell suspension at the concentration of 5×10^5^ /ml was dripped onto glass slides. With the inverted phase contrast microscope, the cells were photographed under 100X magnification. Diameters of more than 1000 cells were manually measured by Image J software, the average value was calculated and compared between LNC and BMMSC, a P value of less than 0.05 was set to be statically significant.

### Microarray Profiling and Data Analysis

A total of 5×10^5^ cells from one culture plate were collected with TRIzol lysis method. 4 replicates were prepared for both the LNC and BMMSC groups. Microarray experiments were performed using Affymetrix Human Primer View Gene Expression Array (CapitalBio Technology, Beijing, China).

Differential expression analysis was performed to highlight transcripts that had at least 2-fold change (FC) in either direction along with a q-value < 0.05 (q-value is the false discovery rate, FDR) when the gene was considered as a differential gene. q-value resembles P-value, and the smaller it is, the more significant the difference is. However, q-value is more reliable when the biological duplicates are more than 3. Enrichment analysis was carried out for the differentially expressed mRNAs via 2 separate pathway databases (Gene Ontology [GO], Kyoto Encyclopedia of Genes and Genomes [KEGG]). P value < 0.05 was considered statistically significantly.

### RT-qPCR Analysis

**Total RNA isolation** 1ml Trizol (Invitrogen, USA) was added to about 5×10^5^ cells. After mixing, 200μl of chloroform was added in and then shaken for 15 seconds and placed on ice for 5 minutes. After centrifugation at 4 °C, 12000 rpm for 15 minutes, the upper aqueous phase was transferred to another new 1.5ml centrifuge tube. Isopropanol was added at equal volume, mixed up and put on ice. Then at 4 °C, the samples were centrifuged at 12000 rpm for 10 minutes, the supernatant discarded, 1ml 75% ethanol (DEPC water mixture) was added and mixed gently. After centrifugation at 4 °C and 7500 rpm for 5minutes, the supernatant was collected and dried at room temperature. 30μL DEPC water was added to dissolve the precipitates and samples collected.

**mRNA reverse transcription to cDNA** The reaction solution is prepared in the 0.2 ml PCR tube according to the Supplementary Table S2. The reverse transcription was performed at 37℃ according to the manufacturer specifications.

**Real-time PCR** The reaction solution was prepared in a 1.5 mL centrifuge tube according to the Supplementary Table S3. The qPCR was performed by initial denaturation at 95 °C for 10 minutes, followed by 40 cycles of 95 °C, 15 seconds for denature, 60℃, 1 minute for annealing, 37 °C for extension. The results were normalized by an internal control, glceraldehyde-3-phosphate dehydrogenase (GAPDH). All assays were performed in triplicate. The relative gene expression was analyzed by the comparative CT method (ΔΔCT). The Gene symbol and Affymetrix ID were detailed in Table 4 and the primer sequences were shown in Supplementary Table S4.

## Results

### LNC Cell Is Smaller than BMMSC

**Figure 1:**
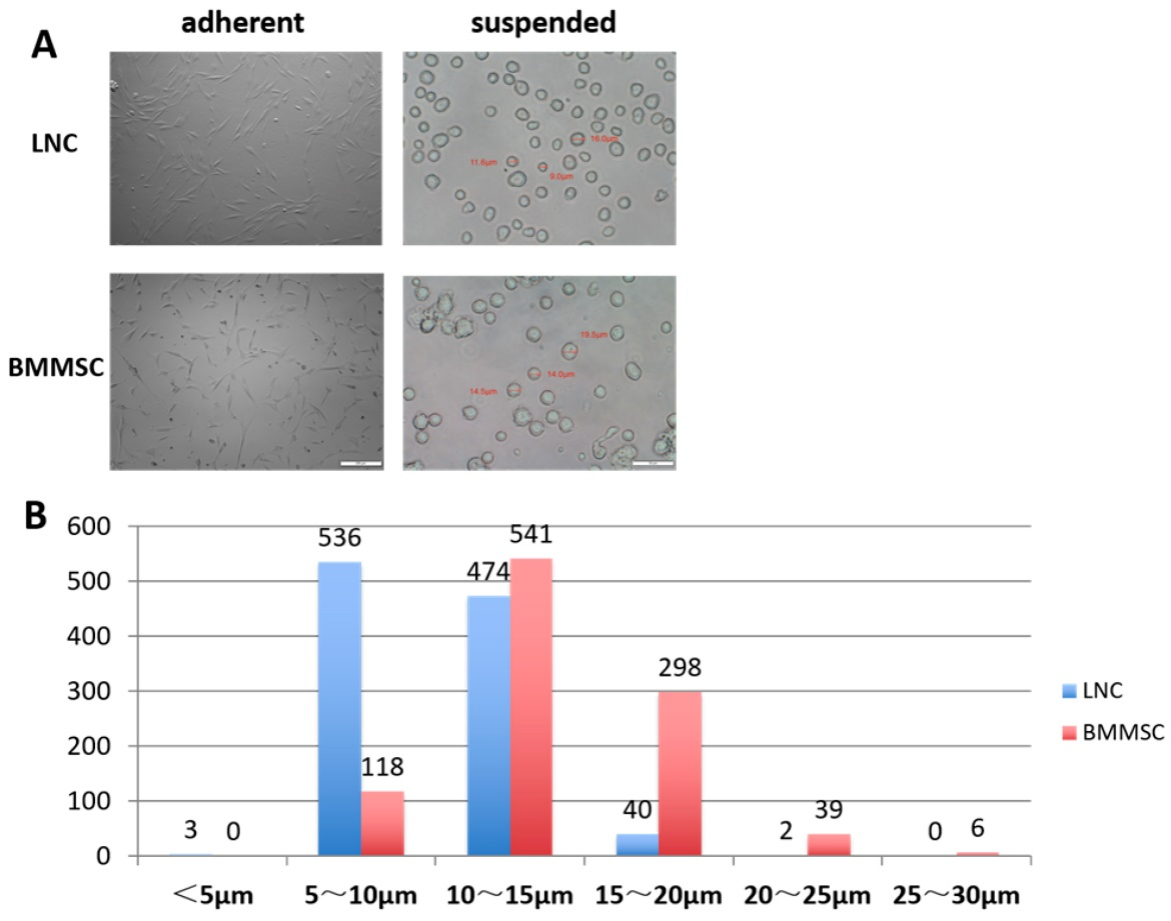
** LNC are spindle cells that smaller than BMMSC.** Cultured on plastic coated with Matrigel, LNC are spindle cells that have two synapses, in contrast, BMMSC are polygonal cells that had three or more synapses (A, left column). Digested with 0.25% trypsin and 1 mM EDTA (T/E) from the culture plate, suspending LNC cells in MESCM medium are round cells smaller than BMMSC (A, right column). Scale Bar of suspension is 50μm and that of adherence is 200μm (A). The distribution characteristics of the diameter of LNC and BMMSC cells is different, with most of the LNC are 5-15μm, while BMMSC are 10-20μm (B).

A total of 1055 LNCs and 1002 BMMSCs were measured. As shown in Table 1, the minimum diameter of LNCs was 4.30 μm, the maximum diameter was 20.5 μm, and 95% of the cells had diameters between 5.6 μm and 14.9 μm (average diameter was 10.2 ±2.4 μm). Comparatively, the minimum diameter of BMMSC was 5.7 μm, the maximum was 27.9 μm and 95% of the BMMSC had diameters between 7.4 μm to 20.6 μm (average diameter was 14.0±3.4 μm). Figure 1A showed that most of the adherent LNC cells had spindle shape with two synapses, while most of the BMMSC cells had polygonal shape with three or more synapses. Figure 1B indicated that most of the LNCs had diameters distributed in 5~10 μm, accounting for 50.8%, while that of BMMSC were 10~15 μm, accounting for 54%. These data suggested that the average size of LNC was smaller than that of BMMSC (P < 0.0001, Fig. 1 and Table 1).

### LNC Expressed Different Genes from BMMSC at the Transcription Level

**Figure 2:**
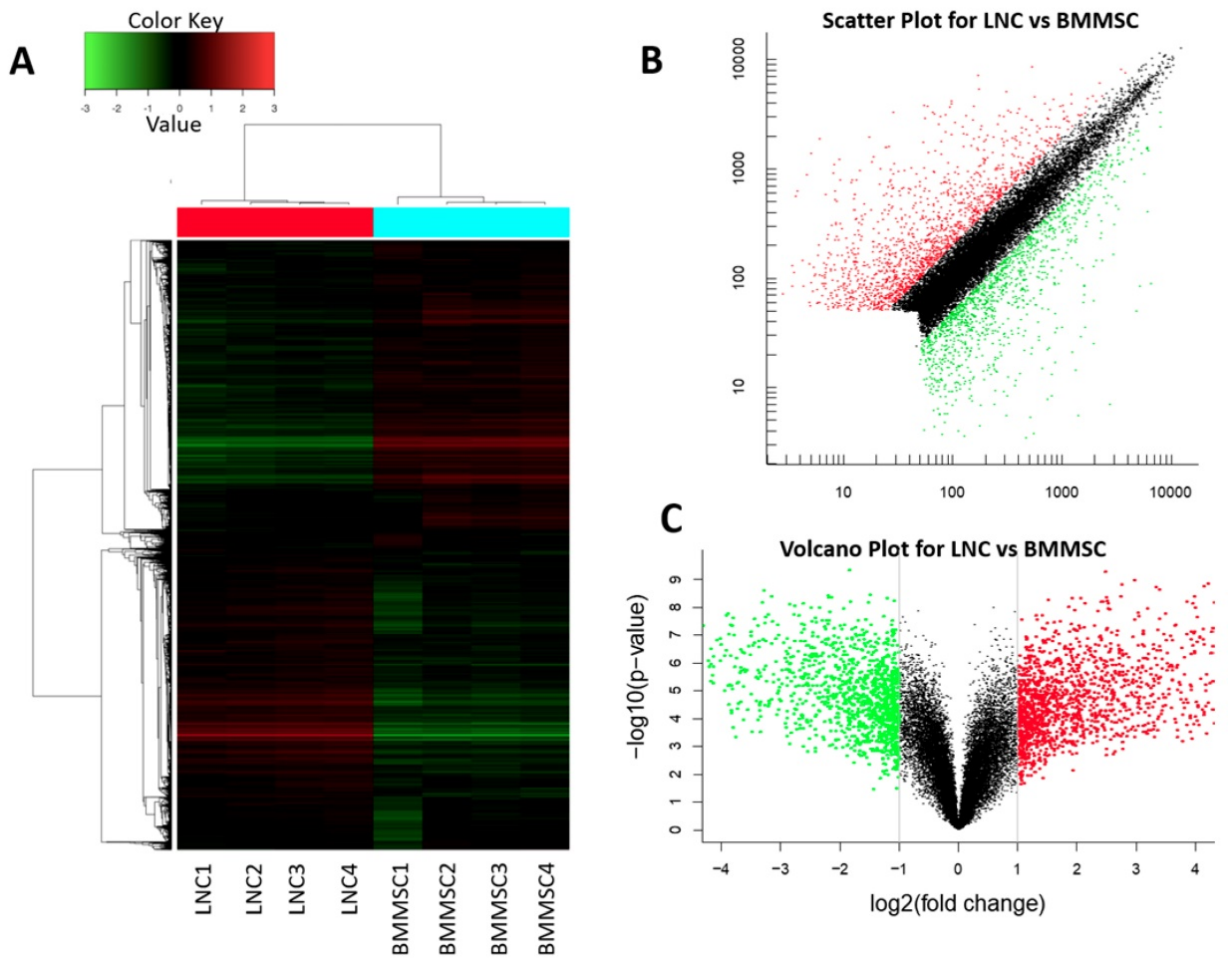
** LNC and BMMSC express genes at different level.** Heat map (A), scatter plot (B) and volcano plot (C) of total transcripts shows 2661 differential expressed genes in which 1468 genes (FC≥2, q<0.05) (red) expressed higher in LNC than in BMMSC and 1193 (FC≤0.5, q<0.05) (green) expressed lower in LNC than BMMSC. Red indicates that gene transcripts in LNC is higher than that in BMMSC, red shows that gene transcripts in BMMSC is higher than that in LNC, while black means that equal in these two cells. From heat map (A), the gene transcription within LNC and BMMSC is consistent.

The data were normalized, and an unbiased data quality control analysis using hierarchical cluster analysis was conducted to illustrate that sample replicates grouped as expected and that there was distinct separation between the sample types (Figure 2A). In total, 20,432 genes (probe, sets) were compared between LNCs and BMMSCs, among which 2,661 genes had fold-change (FC) higher than 2, 349 genes had FC higher than 10, and 13 genes had FC higher than 100 (q value< 0.05, Fig. 2B, Fig. 2C and Table 2).

For the 13 genes with a FC higher than 100-fold, LNCs expressed eight genes higher than BMMSC, namely APCDD1, EGFL6, IGDCC4, GRP, STEAP4, IFI27, FBLN1 and ADH1B, among which APCDD1 was the highest (309-fold), BMMSCs expressed 3 genes higher than LNCs, namely HAPLN1, SLC14A1 and HOXC6 (Table 2), among which HAPLN1 was the lowest (250-fold).

### Enrichment Analysis of Differentially Expressed Genes with GO and KEGG

In order to correlate the biological processes with those genes differentially expressed between LNC and BMMSC, we performed enrichment analyses of the 13 genes with a 100+ FC against GO and KEGG pathways.

GO analysis showed that APCDD1 was related to the WNT pathway, EGFL6 was related to cell cycle, ADH1B was correlated with activity of alcohol dehydrogenase, IGDCC4, GRP and STEAP4 were involved in signal transduction, transport, and protein binding, IFI27 was involved in the process of cell apoptosis, and FBLN1 was involved in extracellular matrix organization.

There are 3 significantly enriched GO pathways, namely extracellular region part (p=2.2e-15), extracellular space (p=5.4e-15), and regulation of Wnt signaling pathway (1.2e-02). The top 1 significantly enriched KEGG pathways were ECM-receptor interaction (p=1.4e-05, Table 3). Differentially expressed genes were also related to the extracellular matrix, growth factors, cytokines, WNT and other selected pathways, as well as some other specific pathways detailed in Table S5.

### Gene Expression Changes Verified by RT-qPCR

**Figure 3:**
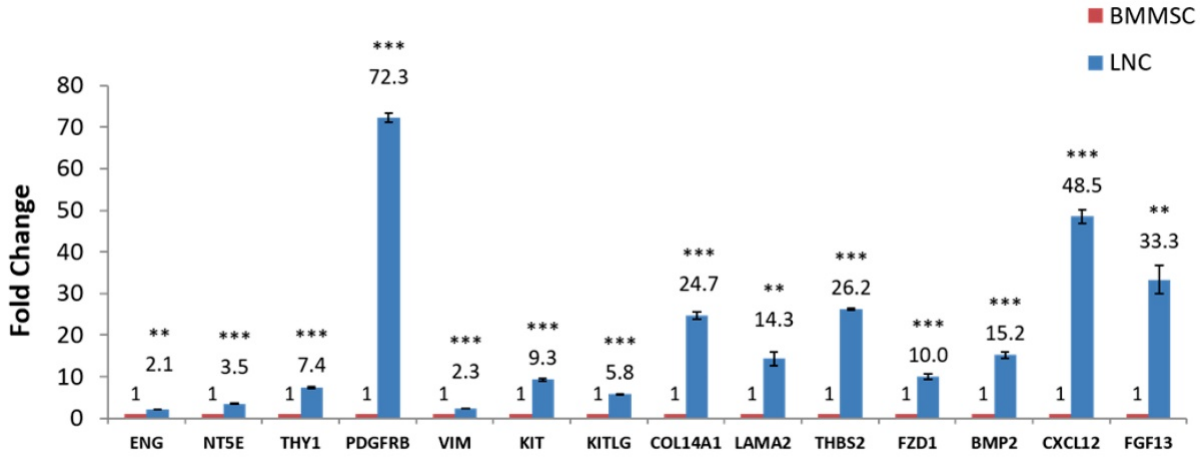
** RT-qPCR confirmation of microarray-detected gene expression difference.** (NY5E [CD73], THY1 [CD90], ENG [CD105], PDGFRβ, Vimentin, KIT [CD117], KITLG [SCF], COL4A1, COL14A1, LAMA1, LAMA2, THBS2, FZD1, BMP2, CXCL12, FGF13 were compared with RT-qPCR using glceraldehyde-3-phosphate dehydrogenase (GAPDH) as internal control. Fold changes shown are statistically significant (P < 0.05). Y axis is 2^-ΔCt^, ^**^P<0.01, ^***^P<0.001.

RT-qPCR was used to quantify expression of some known LNC and BMMSC markers such as CD73, CD90, CD105, PDGFRβ and Vimentin. The results showed that the expression of these genes in LNC was higher than that in BMMSC, among which the FC (LNC vs BMMSC) of CD73 was 3.5, CD90 was 7.4, CD105 was 2.1, and PDGFRβ was 72.4. At the same time, the LNCs expressed SCF at a 5.8 higher level than that of BMMSC. The FC of KIT (CD117), LAMA1, LAMA2, THBS2, FZD1, BMP2, CXCL12, FGF13, COL14A1 were all higher than 2 (P < 0.05) while COL4A1 were lower in LNC, with a FC of 0.2. In addition, the expression of CXCL12 (FC=48.4) and FGF13 (FC=33.1) in LNC was distinctly higher than that of BMMSC (P < 0.05, Fig. 3 and Table 4), which was in consistent with the results of microarray (Table S5).

### Detection of Laminin and Collagen IV with Western blot

**Figure 4:**
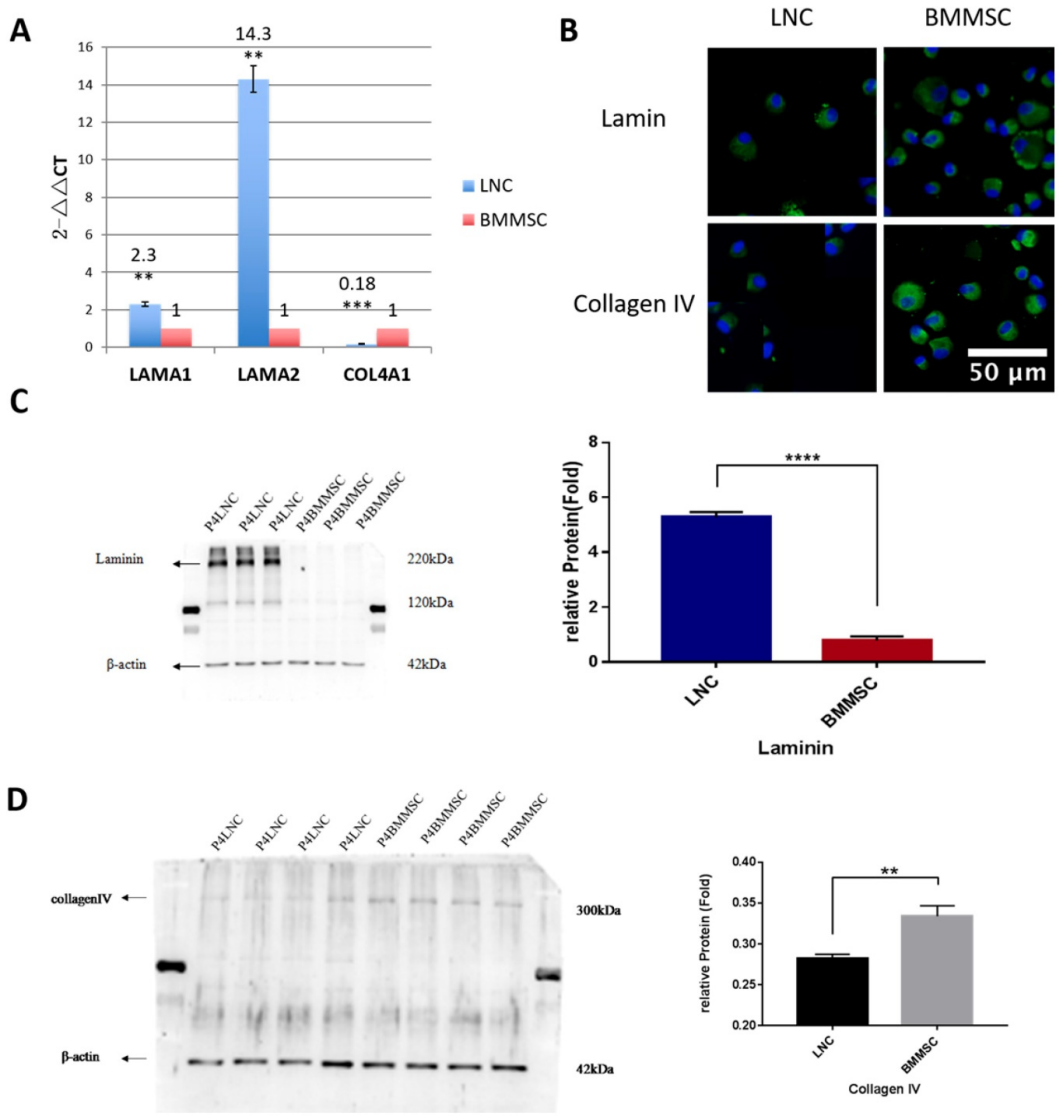
** LNC express more Laminin but less Collagen IV than BMMSC.** To compare the expression level of laminin and collagen IV from transcription and protein level, RT-qPCR was carried out for COL4A1 (coding collagen IV) and LAMA1(coding laminin). Result showed that the LNC express 2.3 folds higher LAMA1 than that of BMMSC (P<0.01), while expression level of COL4A1(FC=0.18) in LNC was lower than BMMSC (P<0.001) (A). Immunofluorescence staining against laminin and collagen IV indicating that both were localized in the surface and plasma of cells (B). Scale Bar 50μm. Western blot showed that LNC expressed 6.7 folds laminin higher than that in BMMSC (P<0.001) (C), while BMMSC expressed 1.2 folds collagen IV higher than that in LNC (P<0.01) (D).

Both LAMA1 and COL4A1 are known to maintain LSC and LNC stemness. To confirm the expression level of LAMA1 (coding laminin) and COL4A1 (coding collagen IV), Western blot was performed. Our data showed that LNC expressed 6.7-fold higher laminin than that in BMMSC (P<0.001) while BMMSC expressed 1.2-fold higher collagen IV than that in LNC (P<0.01, Figure 4), consistent with our RT-qPCR data.

## Discussion

Both LNCs and BMMSCs are pluripotent stem cells derived from stem cell microenvironments. As the first isolated MSC, BMMSCs are well defined for its features, differentiation abilities, and potential clinical applications. In contrast, biological characterization of LNC has not been well understand as was only first reported by Xie 3 in 2012. It has been recognized that these two kinds of cells have many similarities, both of them express CD73, CD90 and CD105 2, 8, 18, can be induced into corneal epithelial like-cells 5, 9, 10, 15, and can prevent LSCD after corneal alkali burn in rats or rabbits when subconjunctivally injected. However, we have reported that LNCs can support LSC better than BMMSC under 3D Matrigel culture environment in vitro 1, 2, and LNCs prevent LSCD caused by alkali burn better than BMMSCs 17. Therefore, exploring the differences between LNC and BMMSC may reveal the key factors for the reconstruction of the LSC niche in vivo.

Herein we observed the morphological differences between LNC and BMMSC in the adherent state. Most of the LNC cells were spindle with two synapses, while BMMSC had more than two synapses (Figure 1). In suspension state, the average diameter of LNC was 10.2± 2.36μm, while that of BMMSC was 14±3.38μm (P < 0.05). As we know, the smaller the cell is, the more primitive it may be 19. Hence, LNCs could be more primitive and pluripotent than BMMSCs 4.

In this study, Genechip was used to compare gene expression profiles between human LNCs and BMMSCs and RT-qPCR was used to verify the Genechip results. Both Genechip and RT-qPCR results showed that the expression of CD73, CD90, CD105, PDGFRβ, Vimentin, SCF and KIT in LNC were higher than that from BMMSC, consistent with our previous reports 2, 17.

Stem cells are regulated in their native niche by a series of adjacent cells, extracellular matrix, and modulating factors sequestered therein^20^.While the cornea limbus, which was universally recognized as the niche of limbal stem cells, is comprised of the extracellular matrix including basement membrane, niche cells such as LNC, blood vessels, stromal cells, nerves, melanocytes and other important components, each one of these components may play different roles in the niche regulation 21, 22. For instance, ECM has been shown to play an important role in the regulation of stem cells and different ECM in *ex vivo* culture system can result in the differential expression of genes 23. For example, 3D Matrigel has been shown to support the stemness of LNC better than coated Matrigel 3, and the conditioned medium containing ECM components promoted wound healing of mice skin 24. Some ECM components that may plan a vital role include EGFL6 and FBLN1. EGFL6 is an extracellular matrix protein that can promote the proliferation of adipose derived stromal vascular cells 25. FBLN1 (Fibulin1) is a secreted glycoprotein that may play a role in cell adhesion and migration and regulates fibronectin-motivated cell junction and diffusion 26. FBLN1 has further been suggested to be involved in eye growth and the formation of myopia 27.

GO analysis show differential expression of genes between LNCs and BMMSCs in various pathways including ECM organization, formats, and regions, and in regulation of WNT singular pathway. KEGG analysis shows the differentially expressed genes participated in ECM-receptors interaction (Table 3). We have discovered that HAPLN1, the lowest gene in LNC (250-fold), is also involved in ECM organization (GO analysis) and APCDD1, the highest gene in LNC (309-fold), is involved in WNT pathway (GO analysis).

Both type IV collagen and laminin are major components of Matrigel, which maintains LSC and LNC stemness 1-3. In our study, we showed LNCs expressed higher laminin but lower collagen IV expression than BMMSCs. The reasons for the differences of expression of laminin and collagen IV between LNCs and BMMSCs need to be further explored. Because upregulation of RNA does not necessarily mean the protein level is also upregulated, we performed Western blotting and confirmed their expression at the protein level. Our results showed that the expression of COL4A1, COL4A2 and COL11A1 in LNC was lower than that of BMMSC, but the expression of LAMA1 and LAMA2 was significantly higher than that of BMMSC (Figure 3, Figure 4, Supplementary Table S5). Hence, we suggest that the high expression of laminin associated genes and the low expression of type IV collagen related genes might account for the fact that LNC support LEPC better than BMMSC.

WNT gene encodes the secreted signal protein. WNT pathway participates in almost all aspects of embryonic development, maintenance of stem cells 28, 29, and regulation of the proliferation and differentiation of MSCs 30. LEPCs cultured without LNCs had upregulatedBMP and WNT pathways, but LEPCs co-cultured with LNCs had inhibited WNT pathway and the WNT suppressor gene DKK1/2 was up-regulated 31. Amniotic membrane extracted HC-HA/PTX3 has also been shown to maintain the quiescence of LEPCs by inhibiting the canonical WNT signaling pathway in LNCs and activating the noncanonical WNT signaling pathway as well as BMP signaling pathway 32.

Our Genechip results show that several genes involved in WNT signaling were expressed differentially between LNC and BMMSC, namely APCDD1, SULF2, DKK2, RSPO3, WNT2 and FZD1 (higher), SOX9 and SFRP1(lower), which have been validated with RT-qPCR. The biggest difference was APCDD1 (309-fold higher in LNCs), an inhibitor of Wnt signaling pathway. The expression of WNT2 and FZD1 in LNC was higher, and the expression of WNT signaling pathway inhibitor SFRP1 was lower, suggesting that WNT signaling pathway in LNC is relatively activated.

In conclusion, although LNC and BMMSC have many similarities, they have dramatically different genes expression related to ECM, WNT signal pathway, and growth factors, which may account for the difference when they serve as niche cells for LSCs. Our results suggest that further analysis of these differential genes may lead to a better understanding for how stem cells are modulated by their niche.

## Supplementary Material

Supplementary tables.

## Figures and Tables

**Table 1:** LNC and BMMSC Cell Diameter

Cell Diameter	LNC	BMMSC
Maximum Value/μm	20.5	27.9
Minimum Value/μm	4.30	5.74
Mean±SD/μm	10.2±2.36	14.0±3.38
95% Confidence Interval/μm	5.58∼14.9	7.35∼20.6
P-value	<0.0001	

**Table 2:** The Differential Gene Expression between LNC and BMMSC (FC≥100 or ≤0.01, q<0.05)

Gene ID	q-value (%)	Fold Change	Gene Title	Gene Symbol	Entrez Gene
11757736_s_at	0	309.1	adenomatosis polyposis coli down-regulated 1	APCDD1	147495
11716532_a_at	0	272.3	"EGF-like-domain, multiple 6"	EGFL6	25975
11746898_x_at	0	189.8	"alcohol dehydrogenase 1B (class I), beta polypeptide"	ADH1B	125
11728991_a_at	0	167.1	"immunoglobulin superfamily, DCC subclass, member 4"	IGDCC4	57722
11756874_a_at	0	152.6	gastrin-releasing peptide	GRP	2922
11720007_a_at	0	132.7	STEAP family member 4	STEAP4	79689
11728992_s_at	0	132.2	"immunoglobulin superfamily, DCC subclass, member 4"	IGDCC4	57722
11757480_x_at	0	115.0	"interferon, alpha-inducible protein 27"	IFI27	3429
11727345_s_at	0	113.7	fibulin 1	FBLN1	2192
11746322_x_at	0	111.6	"alcohol dehydrogenase 1B (class I), beta polypeptide"	ADH1B	125
11727970_a_at	0	0.0074	"solute carrier family 14 (urea transporter), member 1 (Kidd blood group)"	SLC14A1	6563
11740290_a_at	0	0.0069	homeobox C6	HOXC6	3223
11725374_at	0	0.0039	hyaluronan and proteoglycan link protein 1	HAPLN1	1404

**Table 3:** Top Three Enriched Pathways Respectively in GO and KEGG

Pathway	Database	ID	Count	P-Value
Extracellular region part	GO Cellular Component	GO:0044421	99	2.2e-15
Extracellular space	GO Cellular Component	GO:0005615	52	5.4e-15
Wnt signaling pathway	GO Cellular Component	GO:0030111	9	1.2e-02
ECM-receptor interaction	KEGG PATHWAY	hsa04512	9	1.4e-05

**Table 4:** Gene Transcription of Genes Detected with Microarray and RT-qPCR

Gene ID	Gene Symbol	Protein	q-value (%)	Microarray FC	RT-qPCR FC
11715542_s_at	THY1	CD90	0	3.3	7.4
11744681_a_at	NT5E	CD73	0	0.6	3.5
11749246_a_at	ENG	CD105	8.38	1.1	2.1
11715852_at	PDGFRβ	PDGFRβ	0	14.0	72.3
11731394_a_at	VIM	VIM	3.62	1.1	2.3
11728954_a_at	KITLG	SCF	0	1.9	5.8
11721614_a_at	KIT	CD117	0	4.2	9.3
11738028_a_at	LAMA1	the alpha 1 chain of Laminin	0	15.7	2.3
11754429_a_at	LAMA2	the alpha 2 chain of Laminin2 and Laminin4	0	26.7	14.3
11716639_a_at	COL4A1	Collagen type IV alpha 1 chain	0	0.1	0.2
11758810_at	COL14A1	the alpha chain of type XIV collagen	0	5.7	24.7
11742712_a_at	THBS2	member of thrombospondin family	0	10.1	26.2
11739813_a_at	FZD1	Frizzled Class Receptor 1	0	3.4	10
11743497_at	BMP2	ligand of the TGF-beta family	0	5.0	15.2
11720818_a_at	CXCL12	SDF-1	0	15.2	48.5
11720717_a_at	FGF13	member of the fibroblast growth factor (FGF) family	0	5.7	33.3

FC, Fold Change

## References

[1] Li GG, Chen SY, Xie HT, Zhu YT, Tseng SC (2012). Angiogenesis potential of human limbal stromal niche cells. Invest Ophthalmol Vis Sci.

[2] Li GG, Zhu YT, Xie HT, Chen SY, Tseng SC (2012). Mesenchymal stem cells derived from human limbal niche cells. Invest Ophthalmol Vis Sci.

[3] Xie HT, Chen SY, Li GG, Tseng SC (2012). Isolation and expansion of human limbal stromal niche cells. Invest Ophthalmol Vis Sci.

[4] Chen SY, Hayashida Y, Chen MY, Xie HT, Tseng SC (2011). A new isolation method of human limbal progenitor cells by maintaining close association with their niche cells. Tissue Eng Part C Methods.

[5] Acar U, Pinarli FA, Acar DE, Beyazyildiz E, Sobaci G, Ozgermen BB (2015). Effect of allogeneic limbal mesenchymal stem cell therapy in corneal healing: role of administration route. Ophthalmic Res.

[6] Katikireddy KR, Dana R, Jurkunas UV (2014). Differentiation potential of limbal fibroblasts and bone marrow mesenchymal stem cells to corneal epithelial cells. Stem Cells.

[7] Strioga M, Viswanathan S, Darinskas A, Slaby O, Michalek J (2012). Same or not the same? Comparison of adipose tissue-derived versus bone marrow-derived mesenchymal stem and stromal cells. Stem Cells Dev.

[8] Dominici M, Le BK, Mueller I, Slaper-Cortenbach I, Marini F, Krause D (2006). Minimal criteria for defining multipotent mesenchymal stromal cells. The International Society for Cellular Therapy position statement. Cytotherapy.

[9] Jiang TS, Cai L, Ji WY, Hui YN, Wang YS, Hu D (2010). Reconstruction of the corneal epithelium with induced marrow mesenchymal stem cells in rats. Mol Vis.

[10] Rohaina CM, Then KY, Ng AM, Wan Abdul Halim WH, Zahidin AZ, Saim A (2014). Reconstruction of limbal stem cell deficient corneal surface with induced human bone marrow mesenchymal stem cells on amniotic membrane. Transl Res.

[11] Liu H, Zhang J, Liu CY, Hayashi Y, Kao WW (2012). Bone marrow mesenchymal stem cells can differentiate and assume corneal keratocyte phenotype. J Cell Mol Med.

[12] Demirayak B, Yuksel N, Celik OS, Subasi C, Duruksu G, Unal ZS (2016). Effect of bone marrow and adipose tissue-derived mesenchymal stem cells on the natural course of corneal scarring after penetrating injury. Exp Eye Res.

[13] Ma Y, Xu Y, Xiao Z, Yang W, Zhang C, Song E (2006). Reconstruction of chemically burned rat corneal surface by bone marrow-derived human mesenchymal stem cells. Stem Cells.

[14] Ye J, Yao K, Kim JC (2006). Mesenchymal stem cell transplantation in a rabbit corneal alkali burn model: engraftment and involvement in wound healing. Eye (Lond).

[15] Yao L, Li ZR, Su WR, Li YP, Lin ML, Zhang WX (2012). Role of mesenchymal stem cells on cornea wound healing induced by acute alkali burn. PLoS One.

[16] Cejka C, Holan V, Trosan P, Zajicova A, Javorkova E, Cejkova J (2016). The Favorable Effect of Mesenchymal Stem Cell Treatment on the Antioxidant Protective Mechanism in the Corneal Epithelium and Renewal of Corneal Optical Properties Changed after Alkali Burns. Oxid Med Cell Longev.

[17] Li G, Zhang Y, Cai S, Sun M, Wang J, Li S (2018). Human limbal niche cells are a powerful regenerative source for the prevention of limbal stem cell deficiency in a rabbit model. Sci Rep.

[18] Guo P, Sun H, Zhang Y, Tighe S, Chen S, Su CW (2018). Limbal niche cells are a potent resource of adult mesenchymal progenitors. J Cell Mol Med.

[19] Ratajczak MZ, Shin DM, Liu R, Mierzejewska K, Ratajczak J, Kucia M (2012). Very small embryonic/epiblast-like stem cells (VSELs) and their potential role in aging and organ rejuvenation-an update and comparison to other primitive small stem cells isolated from adult tissues. Aging (Albany NY).

[20] Li L, Clevers H (2010). Coexistence of quiescent and active adult stem cells in mammals. Science.

[21] Li W, Hayashida Y, Chen YT, Tseng SC (2007). Niche regulation of corneal epithelial stem cells at the limbus. Cell Res.

[22] Tseng SC, Chen SY, Shen YC, Chen WL, Hu FR (2010). Critical appraisal of ex vivo expansion of human limbal epithelial stem cells. Curr Mol Med.

[23] Pathak M, Olstad OK, Drolsum L, Moe MC, Smorodinova N, Kalasova S (2016). The effect of culture medium and carrier on explant culture of human limbal epithelium: A comparison of ultrastructure, keratin profile and gene expression. Exp Eye Res.

[24] Deng C, He Y, Feng J, Dong Z, Yao Y, Mok H (2017). Extracellular matrix/stromal vascular fraction gel conditioned medium accelerates wound healing in a murine model. Wound Repair Regen.

[25] Oberauer R, Rist W, Lenter MC, Hamilton BS, Neubauer H (2010). EGFL6 is increasingly expressed in human obesity and promotes proliferation of adipose tissue-derived stromal vascular cells. Mol Cell Biochem.

[26] Twal WO, Czirok A, Hegedus B, Knaak C, Chintalapudi MR, Okagawa H (2001). Fibulin-1 suppression of fibronectin-regulated cell adhesion and motility. J Cell Sci.

[27] Li RJ, Ying X, Zhang Y, Ju RJ, Wang XX, Yao HJ (2011). All-trans retinoic acid stealth liposomes prevent the relapse of breast cancer arising from the cancer stem cells. J Control Release.

[28] Clevers H (2006). Wnt/beta-catenin signaling in development and disease. Cell.

[29] Clevers H, Nusse R (2012). Wnt/beta-catenin signaling and disease. Cell.

[30] Ling L, Nurcombe V, Cool SM (2009). Wnt signaling controls the fate of mesenchymal stem cells. Gene.

[31] Han B, Chen SY, Zhu YT, Tseng SC (2014). Integration of BMP/Wnt signaling to control clonal growth of limbal epithelial progenitor cells by niche cells. Stem Cell Res.

[32] Chen SY, Han B, Zhu YT, Mahabole M, Huang J, Beebe DC (2015). HC-HA/PTX3 Purified from Amniotic Membrane Promotes BMP Signaling in Limbal Niche Cells to Maintain Quiescence of Limbal Epithelial Progenitor/Stem Cells.

