# Supplementary material for: Differential Gene Expression between Limbal Niche Progenitors and Bone Marrow Derived Mesenchymal Stem Cells
Source: Int J Med Sci. 2020 Feb 10;17(4):549–57. doi: 10.7150/ijms.40881 (PMC7053302; doi:10.7150/ijms.40881)
Supplement: Supplementary file 1 — Supplementary tables. [file ijmsv17p0549s1.pdf]

# SUPPLEMENTARY MATERIALS

Table S1. Materials Used for Cell Culturing and Isolation

| Materials | Sources | Concentration |
|---|---|---|
| Dulbecco's modified Eagle's Medium (DMEM) | Gibco | DMEM/F-12 (1:1) |
| Knockout Serum Replacement | Gibco | 10% |
| Human Fibroblast Growth Factor-Basic | PEPRO TECH | 4 ng/ml |
| Leukemia inhibitory factor (LIF) | PEPRO TECH | 10 ng/ml |
| Insulin-Transferrin-sodium selenite media supplement | Sigma | 5 µg/ml insulin, 5 µg/ml Transferrin, 5 ng/ml sodium selenite |
| Amphotericin B | Nanjing Sai Hong Rui Biotechnology Co., Ltd | 50 µg/ml |
| Gentamicin | YiChang Humanwell | 1.25 µg/ml |
| Collagenase A | Invitrogen | 1 mg/ml |
| Matrigel™ Basement Membrane Matrix | BD Biosciences | 5% |
| Trypsin and EDTA (T/E) | Servicebio | 0.25% and 1 mM |
| Dimethyl Sulfoxide | Shanghai Xihe Technology Co., Ltd | 0.5% |
| Human PrimerView Gene Expression Array | Affymetrix, Beijing, China | N/A |
| OriCell$^{TM}$ Human Mesenchymal Stem Cells（Derived from bone marrow） | Cyagen, Guangzhou, China | N/A |
| OriCell$^{TM}$ Human Mesenchymal Stem Cell Growth Medium | Cyagen, Guangzhou, China | N/A |

Table S2 Reaction system of RNA reverse to cDNA

| Component | Vol. (μl) |
|---|---|
| 5x RT Buffer | 4 |
| Enzyme Mix | 1 |
| Primer Mix | 1 |
| RNA template(0.5μg) + H2O | 14 |
| Total volume | 20 |

Table S3 Reaction system of real time RT-qPCR

| Component | Vol. |
| --- | --- |
| 2×SYBR Green qPCR buffer | 5 μl |
| Forward primer (10μ M) | 0.5 μl |
| Reverse primer (10μ M) | 0.5 μl |
| Template | 5 ng |
| $ddH_2O$ | X μl |
| Total volume | 10 μl |

Table S4. Primer Sequence Use for Real-time RT-qPCR

| Gene Bank NO. | Primer | Sequence(5'to3') |
|---|---|---|
| ENG | Forward | CTGACCTGTCTGGTTGCACAA |
| | Reverse | GATGAGGAAGGCACCAAAGG |
| NT5E | Forward | AATCACTGCATTACAACCTGAAGTAGA |
| | Reverse | CTTTCTGAGCGATGAGTTTATCCA |
| THY1 | Forward | AGAGGGCTACAAAGGAGGACAA |
| | Reverse | ACTCCACCCCACCTGACTTG |
| PDGFRB | Forward | GGCACCAACCCTGCATTG |
| | Reverse | CTCCCTCCTTGGACCAACTCT |
| VIM | Forward | TGCCAACCGGAACAATGAC |
| | Reverse | ACTGCACCTGTCTCCGGTACTC |
| KIT | Forward | TATTCCCAAGCCCATGAGTC |
| | Reverse | ACGTGGAACACCAACATCCT |
| KITLG | Forward | CAGTCATAGATTGGAGTTTGCAT |
| | Reverse | CACATACACATCACATTTTACAACCT |
| SOX4 | Forward | GGCAGTTCCGTTAAGGGTTT |
| | Reverse | TGTTTTTGTGGCCTTGAATTT |
| COL4A1 | Forward | TGGCTCTGGCTGTGGCAAA |
| | Reverse | CCCAATGACACCTTGTAACCC |
| COL14A1 | Forward | GCTCAAAGAGGAATTGGGAAA |
| | Reverse | TTCTTTTCCTGCTAGGGACAT |
| LAMA1 | Forward | GCCAGCTCTAATGCCATC |
| | Reverse | GGGTTGACAAATTCCTCCAAA |
| LAMA2 | Forward | GCAAGCCACTGGAGGTTAAT |
| | Reverse | GACAGACTCTTCCTGGGGTTA |

| | | |
|---|---|---|
| THBS2 | Forward | AAACCCAAGTGCCTTCAGAG |
| | Reverse | GCCCAATTTTCACTCCACAT |
| FZD1 | Forward | GAGATAGCGCCTGAAATAAACAAAA |
| | Reverse | GAGGAGCACACAGGATTTAATGC |
| BMP2 | Forward | AGAAAGAATAAAGCAGGATCCATAGAA |
| | Reverse | GGAAAGAAGAACAACAAACCATCA |
| CXCL12 | Forward | TGCTTACCCGCAAAAGACAA |
| | Reverse | GGCTTCAGAGGCAATCACAAA |
| FGF13 | Forward | TGTCAACCCCAGTCAGTAAAGA |
| | Reverse | TTTACACAAGAAGCCACAACAA |
| GAPDH | Forward | TGACTTCAACAGCGACACCCA |
| | Reverse | CACCCTGTTGCTGTAGCCAAA |

Table S5. Differential gene expression between LNC and BMMSC in ECM and growth factor related pathways, as well as WNT and other selected pathways

| Gene ID | q-value (%) | Fold Change | Gene Title | Gene Symbol | Entrez Gene |
|---|---|---|---|---|---|
| extracellular matrix organization | | | | | |
| 11716532_a_at | 0 | 272.3 | "EGF-like-domain, multiple 6" | EGFL6 | 25975 |
| 11727345_s_at | 0 | 113.79 | fibulin 1 | FBLN1 | 2192 |
| 11723635_s_at | 0 | 35.7 | "matrix metallopeptidase 3 (stromelysin 1, progelatinase)" | MMP3 | 4314 |
| 11740238_a_at | 0 | 34.3 | sulfatase 2 | SULF2 | 55959 |
| 11730726_s_at | 0 | 33.4 | "angiotensinogen (serpin peptidase inhibitor, clade A, member 8)" | AGT | 183 |
| 11721475_a_at | 0 | 32.0 | glycoprotein M6B | GPM6B | 2824 |
| 11754429_a_at | 0 | 26.7 | "laminin, alpha 2" | LAMA2 | 3908 |
| 11729385_at | 0 | 24.8 | forkhead box F1 | FOXF1 | 2294 |
| 11723030_at | 0 | 16.8 | cysteine-rich secretory protein LCCL domain containing 2 | CRISPLD2 | 83716 |
| 11715496_a_at | 0 | 16.0 | cathepsin K | CTSK | 1513 |
| 11738028_a_at | 0 | 15.8 | "laminin, alpha 1" | LAMA1 | 284217 |
| 11756706_a_at | 0 | 12.6 | dipeptidyl-peptidase 4 | DPP4 | 1803 |
| 11739782_a_at | 0 | 11.6 | "synuclein, alpha (non A4 component of amyloid precursor)" | SNCA | 6622 |
| 11746856_a_at | 0 | 0.054 | "serpin peptidase inhibitor, clade E (nexin, | SERPINE1 | 5054 |

| | | | | | |
|---|---|---|---|---|---|
| | | | plasminogen activator inhibitor type 1), member 1" | | |
| 11725766_x_at | 0 | 0.078 | "collagen, type XI, alpha 1" | COL11A1 | 1301 |
| 11717803_a_at | 0 | 0.096 | netrin 4 | NTN4 | 59277 |
| 11757425_s_at | 0 | 0.071 | vascular cell adhesion molecule 1 | VCAM1 | 7412 |
| 11720448_at | 0 | 0.095 | SRY (sex determining region Y)-box 9 | SOX9 | 6662 |
| 11715453_a_at | 0 | 0.091 | "collagen, type IV, alpha 2" | COL4A2 | 1284 |
| 11726855_at | 0 | 0.077 | "ADAM metallopeptidase with thrombospondin type 1 motif, 5" | ADAMTS 5 | 11096 |
| 11716639_a_at | 0 | 0.097 | "collagen, type IV, alpha 1" | COL4A1 | 1282 |
| 11725584_at | 0 | 0.086 | hyaluronan synthase 3 | HAS3 | 3038 |
| 11718243_a_at | 0 | 0.081 | "integrin, alpha 7" | ITGA7 | 3679 |
| 11724065_at | 0 | 0.074 | integrin-binding sialoprotein | IBSP | 3381 |
| 11727790_x_at | 0 | 0.044 | secreted phosphoprotein 1 | SPP1 | 6696 |
| 11725376_at | 0 | 0.011 | hyaluronan and proteoglycan link protein 1 | HAPLN1 | 1404 |
| ECM-receptor interaction | | | | | |
| 11754429_a_at | 0 | 26.7 | "laminin, alpha 2" | LAMA2 | 3908 |
| 11738028_a_at | 0 | 15.7 | "laminin, alpha 1" | LAMA1 | 284217 |
| 11742712_a_at | 0 | 10.1 | thrombospondin 2 | THBS2 | 7058 |
| 11725766_x_at | 0 | 0.078 | "collagen, type XI, alpha 1" | COL11A1 | 1301 |

| | | | | | |
|---|---|---|---|---|---|
| 11715453_a_at | 0 | 0.091 | "collagen, type IV, alpha 2" | COL4A2 | 1284 |
| 11716639_a_at | 0 | 0.097 | "collagen, type IV, alpha 1" | COL4A1 | 1282 |
| 11718243_a_at | 0 | 0.081 | "integrin, alpha 7" | ITGA7 | 3679 |
| 11724065_at | 0 | 0.074 | integrin-binding sialoprotein | IBSP | 3381 |
| 11727790_x_at | 0 | 0.044 | secreted phosphoprotein 1 | SPP1 | 6696 |
| Focal adhesion | | | | | |
| 11754429_a_at | 0 | 26.7 | "laminin, alpha 2" | LAMA2 | 3908 |
| 11722855_at | 0 | 19.5 | platelet derived growth factor D | PDGFD | 80310 |
| 11738028_a_at | 0 | 15.7 | "laminin, alpha 1" | LAMA1 | 284217 |
| 11715852_at | 0 | 14.0 | "platelet-derived growth factor receptor, beta polypeptide" | PDGFRB | 5159 |
| 11742712_a_at | 0 | 10.1 | thrombospondin 2 | THBS2 | 7058 |
| 11725766_x_at | 0 | 0.078 | "collagen, type XI, alpha 1" | COL11A1 | 1301 |
| 11715453_a_at | 0 | 0.091 | "collagen, type IV, alpha 2" | COL4A2 | 1284 |
| 11716639_a_at | 0 | 0.097 | "collagen, type IV, alpha 1" | COL4A1 | 1282 |
| 11718243_a_at | 0 | 0.081 | "integrin, alpha 7" | ITGA7 | 3679 |
| 11724065_at | 0 | 0.074 | integrin-binding sialoprotein | IBSP | 3381 |
| 11727790_x_at | 0 | 0.044 | secreted phosphoprotein 1 | SPP1 | 6696 |
| growth factor activity | | | | | |
| 11756003_x_at | 0 | 74.8 | insulin-like growth factor 2 (somatomedin A) /// INS-IGF2 readthrough | IGF2 /// INS-IGF2 | 3481 /// 72396 |

| | | | | | |
|---|---|---|---|---|---|
| | | | | | 1 |
| 11723033_at | 0 | 46.0 | nephroblastoma overexpressed | NOV | 4856 |
| 11730726_s_at | 0 | 33.4 | "angiotensinogen (serpin peptidase inhibitor, clade A, member 8)" | AGT | 183 |
| 11722855_at | 0 | 19.5 | platelet derived growth factor D | PDGFD | 80310 |
| 11720818_a_at | 0 | 15.2 | chemokine (C-X-C motif) ligand 12 | CXCL12 | 6387 |
| 11758619_s_at | 0 | 0.038 | leukemia inhibitory factor | LIF | 3976 |
| 11751872_x_at | 0 | 0.091 | epithelial mitogen | EPGN | 25532 4 |
| 11734363_at | 0 | 0.082 | growth differentiation factor 6 | GDF6 | 39225 5 |
| 11756560_a_at | 0 | 0.029 | Norrie disease (pseudoglioma) | NDP | 4693 |
| response to growth factor | | | | | |
| 11721895_x_at | 0 | 39.2 | growth arrest-specific 1 | GAS1 | 2619 |
| 11740238_a_at | 0 | 34.3 | sulfatase 2 | SULF2 | 55959 |
| 11730726_s_at | 0 | 33.4 | "angiotensinogen (serpin peptidase inhibitor, clade A, member 8)" | AGT | 183 |
| 11715852_at | 0 | 14.0 | "platelet-derived growth factor receptor, beta polypeptide" | PDGFRB | 5159 |
| 11739782_a_at | 0 | 11.6 | "synuclein, alpha (non A4 component of amyloid precursor)" | SNCA | 6622 |
| 11719292_a_at | 0 | 11.2 | integrin beta 1 binding protein 1 | ITGB1BP 1 | 9270 |
| 11746856_a_at | 0 | 0.054 | "serpin peptidase | SERPINE | 5054 |

| | | | | | |
|---|---|---|---|---|---|
| | | | inhibitor, clade E (nexin, plasminogen activator inhibitor type 1), member 1" | 1 | |
| 11757425_s_at | 0 | 0.071 | vascular cell adhesion molecule 1 | VCAM1 | 7412 |
| 11720448_at | 0 | 0.095 | SRY (sex determining region Y)-box 9 | SOX9 | 6662 |
| 11715453_a_at | 0 | 0.091 | "collagen, type IV, alpha 2" | COL4A2 | 1284 |
| 11723246_s_at | 0 | 0.097 | secreted frizzled-related protein 1 | SFRP1 | 6422 |
| 11754706_a_at | 0 | 0.084 | hedgehog interacting protein | HHIP | 64399 |
| 11724065_at | 0 | 0.074 | integrin-binding sialoprotein | IBSP | 3381 |
| 11737331_at | 0 | 0.071 | "phosphodiesterase 1C, calmodulin-dependent 70kDa" | PDE1C | 5137 |
| 11719916_at | 0 | 0.067 | "interleukin 1, beta" | IL1B | 3553 |
| 11726023_a_at | 0 | 0.039 | endothelin 1 | EDN1 | 1906 |
| 11731445_at | 0 | 0.018 | ankyrin repeat domain 1 (cardiac muscle) | ANKRD1 | 27063 |
| regulation of Wnt signaling pathway | | | | | |
| 11757736_s_at | 0 | 309.18 | adenomatosis polyposis coli down-regulated 1 | APCDD1 | 147495 |
| 11740238_a_at | 0 | 34.31 | sulfatase 2 | SULF2 | 55959 |
| 11728088_at | 0 | 28.7 | dickkopf WNT signaling pathway inhibitor 2 | DKK2 | 27123 |
| 11724619_at | 0 | 21.1 | R-spondin 3 | RSPO3 | 84870 |
| 11744512_a_at | 0 | 14.0 | TraB domain containing 2A | TRABD2A | 129293 |

| | | | | | |
|---|---|---|---|---|---|
| 11754365_s_at | 0 | 14.0 | receptor tyrosine kinase-like orphan receptor 2 | ROR2 | 4920 |
| 11756162_s_at | 0 | 13.3 | phosphatidic acid phosphatase type 2B | PPAP2B | 8613 |
| 11731558_a_at | 0 | 6.1 | wingless-type MMTV integration site family member 2 | WNT2 | 7472 |
| 11739813_a_at | 0 | 3.4 | frizzled class receptor 1 | FZD1 | 8321 |
| 11720448_at | 0 | 0.095 | SRY (sex determining region Y)-box 9 | SOX9 | 6662 |
| 11723246_s_at | 0 | 0.097 | secreted frizzled-related protein 1 | SFRP1 | 6422 |
| mesenchyme development | | | | | |
| 11723006_a_at | 0 | 42.5 | S100 calcium binding protein A4 | S100A4 | 6275 |
| 11726023_a_at | 0 | 0.039 | endothelin 1 | EDN1 | 1906 |
| 11747223_a_at | 0 | 12.9 | endothelin receptor type B | EDNRB | 1910 |
| 11723246_s_at | 0 | 0.097 | secreted frizzled-related protein 1 | SFRP1 | 6422 |
| 11729385_at | 0 | 24.8 | forkhead box F1 | FOXF1 | 2294 |
| 11721392_a_at | 0 | 0.080 | lysyl oxidase-like 3 | LOXL3 | 84695 |
| 11715852_at | 0 | 14.0 | "platelet-derived growth factor receptor, beta polypeptide" | PDGFRB | 5159 |
| 11720447_s_at | 0 | 0.168 | SRY (sex determining region Y)-box 9 | SOX9 | 6662 |
| Cytokine-cytokine receptor interaction | | | | | |
| 11715852_at | 0 | 14.0 | "platelet-derived growth factor receptor, beta polypeptide" | PDGFRB | 5159 |

| | | | | | |
|---|---|---|---|---|---|
| 11718025_at | 0 | 11.5 | "interleukin 1 receptor, type I" | IL1R1 | 3554 |
| 11719916_at | 0 | 0.067 | "interleukin 1, beta" | IL1B | 3553 |
| 11722598_s_at | 0 | 23. 6 | atypical chemokine receptor 3 | ACKR3 | 57007 |
| 11758608_s_at | 0 | 0.028 | "colony stimulating factor 2 receptor, beta, low-affinity (granulocyte-macrophage )" | CSF2RB | 1439 |
| 11720818_a_at | 0 | 15.2 | chemokine (C-X-C motif) ligand 12 | CXCL12 | 6387 |
| 11728039_s_at | 0 | 26.5 | chemokine (C-C motif) ligand 8 | CCL8 | 6355 |
| 11758619_s_at | 0 | 0.038 | leukemia inhibitory factor | LIF | 3976 |
